# Implementation of the Strategy for Breastfeeding and Complementary Feeding in the Federal District in Brazil

**DOI:** 10.3390/ijerph19095003

**Published:** 2022-04-20

**Authors:** Amanda Souza Moura, Muriel Bauermann Gubert, Sonia Isoyama Venancio, Gabriela Buccini

**Affiliations:** 1 Department of Nutrition, Center of Epidemiological Studies of Health and Nutrition (NESNUT), Faculty of Health Sciences, University of Brasília, Brasília 70910-900, Brazil; murielgubert@gmail.com; 2 Institute of Health, State Secretariat of São Paulo Health, São Paulo 01314-000, Brazil; soniavenancio@uol.com.br; 3 Department of Social and Behavioral Health, University of Nevada, Las Vegas, NV 89119, USA; gabriela.buccini@unlv.edu

**Keywords:** breastfeeding, complementary feeding, health assessment, primary health care

## Abstract

Background: The Brazilian Breastfeeding and Complementary Feeding Strategy—Estratégia Amamenta e Alimenta Brasil (EAAB) aims to promote optimal breastfeeding (BF) and complementary feeding (CF) practices through the training of primary health professionals. Competition among health priorities and programs is one of the organizational contextual barriers to consolidating the implementation of the EAAB. Methods: This case study included six Primary Health Units (PHU) certified in the EAAB. Documentary analysis, interviews, and surveys were conducted, which informed a conceptual logical model. Organizational context indicators (positive and negative) were identified across the logical model based on the Matus Triangle, and they were used to analyze the degree of implementation of the EAAB in the PHUs. Results: The logic model elucidated six stages of EAAB implementation, but none on post-certification monitoring. Ten indicators positively influenced the implementation, including having legislation that prioritizes BF and CF. Seven indicators exerted negative influence, especially the lack of specific funding resources for the EAAB. Only one PHU had a consolidated degree of implementation. Conclusions: Lack of specific funding, monitoring of BF and CF practices, and compliance with certification criteria are the main challenges for the EAAB’s sustainability.

## 1. Introduction

The World Health Organization (WHO) recommends exclusive breastfeeding (EBF) during the first six months of life and complementary feeding (CF) with the introduction of healthy foods beginning at six months and continued for up to two years or more [1,2,3]. Breastfeeding (BF) reduces the chances of children developing diseases in childhood, such as infections and diarrhea [3,4], and other diseases in adulthood, including diabetes and obesity [3,5]. Furthermore, BF favors the adequate development of the oral cavity, teeth, and speech and has positives effects on intelligence and on achieving higher levels of income in adult life [3,6,7]. For the woman, breastfeeding also brings benefits, reducing her chances of developing diabetes and breast, ovary, and uterus cancer [3,6]. At six months old, along with BF, the introduction of adequate and healthy complementary food helps to prevent overweight and nutritional deficiencies, such as vitamin A and iron deficiencies, for better childhood growth and development. A healthy introduction to CF also benefits the formation of eating habits [3].

Policies and actions developed within the Brazilian Unified Health System (SUS) to promote recommended BF and CF practices include the National Policy of Food and Nutrition (PNAN—Política Nacional de Alimentação e Nutrição) [7] and the National Policy on Integral Attention to the Health of the Child (PNAISC—Política Nacional de Atenção Integral à Saúde da Criança) [8]. Under the PNAISC umbrella, several actions are included, such as the Baby-Friendly Hospital Initiative (BFHI) [8,9], the Action to Support Working Women Who Breastfeed (MTA—Mulher Trabalhadora que Amamenta) [8,9], the Brazilian Marketing Code for Food, Nipples, Pacifiers, and Bottles for Infants and Young Children (NBCAL—Norma Brasileira de Comercialização de Alimentos para Lactentes), the Food Guide for Brazilian Children Under Two Years Old (Guia Alimentar para Crianças Brasileiras Menores de Dois Anos), the National Day of Human Milk Donation (Dia Nacional de Doação de Leite Humano), and the largest Network of Human Milk Banks (Rede Banco de Leite Humano) globally [9].

The investment in this large portfolio of policies, programs, and actions to promote BF and CF in the first two years of life has generated important advances in the prevalence of EBF in children under six months old in Brazil, which rose from 2.9% in 1986 to 45.7% in 2019 [10]. Total BF in children under 24 months increased from 37.4% in 1986 to 60.9% in 2019 [10]. Nevertheless, the prevalence of EBF is lower than the 50% established by the 2025 WHO Global Nutrition Target [2]. Likewise, CF is still far from ideal in Brazilian children aged six to eleven months, where despite the high consumption of vegetables (71.2%), meat and eggs (61.5%), and beans (66.4%) [9], there is a high and nonrecommended consumption of other foods such as porridge (55.2%) [9], soft drinks (40.5%), fried foods (39.4%), snacks (39.4%), and sweets (37.8%) [10]. The data indicate the need to invest in continued actions to support the practice of BF and FC, through individual and collective counseling carried out by health professionals within the PHU.

Counseling by qualified health professionals about the feeding of infants and young children increases women’s knowledge and confidence, as well as the practice of BF and CF [11,12,13]. Community BF counseling is a proven effective intervention to increase EBF rates [14]. Hence, the Brazilian Breastfeeding and Complementary Feeding Strategy (EAAB—Estratégia Amamenta e Alimenta Brasil) aims to train primary health professionals to support BF and CF by increasing their knowledge based on a reflection of their work reality. Based on the principles of continuing education through structured professional training workshops and using critical reflective methodologies, the EAAB aims to create spaces for discussion and learning among primary health professionals [15]. PHUs implementing the EAAB are encouraged to comply with six preestablished certification criteria [9,15,16]: (1) develop systematic individual or collective actions to promote BF and CF; (2) monitor BF and CF rates; (3) have an instrument for child health care organization (flowchart, map, protocol, line of care, or other) to detect problems related to BF and CF; (4) comply with the Brazilian Guidelines for the Marketing of Food for Infants and Young Children, Nipples, Pacifiers, and Bottles (NBCAL) and with Law No. 11265 of 2006, and do not distribute “substitutes” for breast milk at the PHU; (5) count on the participation of at least 85% of the team’s professionals in the developed workshops, and (6) fulfill at least one BF action and one CF action established in the action plan [15]. Therefore, PHUs certified in the EAAB are considered to provide the highest standard of support in BF and CF.

There have been some challenges to implementing the EAAB. According to the EAAB guidelines, one of the tasks of the tutor (PHU health professional trained at the EAAB to disseminate it) is to train the PHU in a six-hour workshop and continuously support it to meet the certification criteria [16]. However, according to data from the Brazilian Ministry of Health, around 4901 professionals were trained as tutors by March 2018 to disseminate the EAAB; however, only 3127 PHUs were trained by these tutors and only 109 PHUs were certified [17]. This discrepant scenario between the number of tutors, trained PHUs, and certified PHUs was also found in another Program similar to EAAB and that preceded it, the Brazil Breastfeeding Network [18]. In this program, some aspects were identified that hindered its implementation process (which ranged from the training of professionals as tutors to the certification of PHUs, such as in the EAAB), such as the low prioritization of the program in the management plans (state and municipal), the competition with other projects and programs, and the lack of financial resources to make it viable [19]. In addition, the high turnover of professionals in the trained PHUs is also a factor identified both in the Brazil Breastfeeding Network and in the current EAAB, which can also make it difficult to comply with the certification criteria [20,21,22]. In the EAAB, there is no requirement for a monitoring system after certification and, consequently, little is known about sustainability after certification [15].

Furthermore, the EAAB does not require any monitoring system after certification and, consequently, little is known about sustainability after certification [16]. Our hypothesis is that the assessment of the organizational context can help to identify the degree of implementation and barriers to make public health policies and programs more efficient [13,19]. Thus, our objective was to evaluate the influence of the organizational context on the degree of implementation of the EAAB in certified PHUs in the Federal District, Brazil.

## 2. Materials and Methods

### 2.1. Study Setting

The Federal District (FD) is the capital of Brazil and is located in the Central-Western region of the country. The FD has management attributions of a state and municipality. In 2019, the FD population was estimated at 3,012,718 inhabitants [23], with just over 10% of the population living below the poverty line [24]. In 2018, 63.9% of the population did not have a private health insurance plan; therefore, they rely on the PHUs to access health care [25]. In addition, in 2018, 44,195 live births were registered in the Live Birth Information System (SINASC) in relation to the FD [26]. Specifically, the Federal District began implementation of the EAAB in 2013 [17].

The EAAB is implemented by the three spheres of Brazilian management: federal, state, and municipal. At the federal level, coordination is done by the Ministry of Health, which prepares technical materials on the EAAB, provides an online monitoring system for the EAAB and BF and CF indicators, in addition to certifying the PHUs that meet the six EAAB certification criteria. The state and municipal levels have a figure of coordination (state coordinator and municipal coordinator), usually a professional linked to the health department who is responsible for coordinating the activities of the EAAB related to their level of management, such as the training of tutors, monitoring of the PHU (trained in workshops of six hours), and guidance on the use of monitoring systems that the Ministry of Health makes available. There is also the figure of the tutor, a health professional who is trained in an EAAB workshop (necessary to obtain this designation), who has the competence to train the entire team of professionals at the PHU (in a six-hour workshop) to develop activities aimed at promoting BF and healthy CF. After the six-hour workshop, the tutor is responsible for supporting the PHU in building an action plan on activities related to BF and healthy CF, as well as carrying out complementary activities, if necessary, to help the PHU meet the EAAB certification criteria. The PHU also has a manager, who is a professional formally designated to manage the health service of the PHU and who must also be involved in the six-hour workshop [16].

In 2018, the FD had 131 PHUs, of which 31 were trained at the EAAB (most received the six-hour workshop but did not meet the EAAB certification criteria). Only six were certified by the EAAB (received the six-hour workshop and met EAAB certification criteria) [17].

### 2.2. Study Design

This is a case study of the implementation of EAAB in the FD. A case study design has been considered an appropriate design for evaluative research on implementation, especially to identify a phenomenon within a context [27]. A type 1 implementation analysis methodology following the four steps adapted by Venancio et al. (2013) [19] was used to identify the effect of the organizational context on the degree of program implementation [28]: (1) create the conceptual logical model of the EAAB in the FD; (2) evaluate the organizational context; (3) evaluate the degree of implementation of the EAAB in the Health Units, and (4) analyze the influence of the organizational context on the degree of implementation.

### 2.3. Analyses

#### 2.3.1. Create the Conceptual Logical Model of the EAAB in the FD

Documentation review included three documents published by the Brazilian Ministry of Health related to the implementation of the EAAB: (1) Ordinance No. 1920 of 5 September 2013, which established the EAAB [15]; (2) the EAAB Implementation Manual [16], and (3) the Instruction for the Implementation Plan of the EAAB [29]. The analysis of these documents informed the development of a conceptual logic model of EABB implementation in the FD.

Specifically, information about the implementation stages of the EAAB was taken from Ordinance No. 1920 [15], as well as the responsibilities and capacities explicitly established for the state and municipal context, considering that the FD has these two attributions in its management. The Implementation Manual [16] and the Instructions for the Implementation Plan of the EAAB [29] also identified and detailed the responsibilities and capacities established for the context of the FD, as well as information on the implementation stages and the actors involved. With this information, a hierarchical logical model was developed, describing the general capacities of the FD in the EAAB, delimiting the implementation stages of the EAAB in the FD, and describing the capacities defined for the FD in each of them.

#### 2.3.2. Evaluate the Organizational Context

The analysis of the organizational context employed the theoretical reference model of the Triangle of Government developed by Matus, consisting of three categories: (1) Government Project, (2) Government Capacity, and (3) System Governability [30]. These categories have been previously adapted to the context of the EAAB [19], resulting in a total of 17 indicators.

The Government Project category included seven indicators related to interest, proposals, policies, and actions directed and/or prioritized by the management in relation to PHC, BF, and CF, and/or the EAAB, as well as the allocation of financial resources. The indicators analyzed in this category were Family Health Strategy coverage (above 50%) and presence/absence of some conditions, such as BF and CF as a priority in policy/legislation or guideline, interest in the implementation of the EAAB, positive receptivity of the EAAB in the sphere of government, competition of the EAAB with other priorities, existence of activities, actions, and/or programs complementary to the EAAB, and specific financial resource for the EAAB.

The Government Capacity category included six indicators that assessed the existence of a manager and/or team, as well as the theoretical capacity and technical experience accumulated by the management and team in conducting and administering the EAAB, the ability to put the planned actions into operation, and the use of management technologies to monitor the action. The indicators measured in this category were existence of an area for children’s health; existence of an EAAB Coordinator for BF and CF actions; stability of the person responsible for BF and healthy CF actions (bonded); professional experience of the person responsible for the actions of BF and healthy CF; institutional roles of the EAAB managers compatible with the organization chart; use of one or more management technologies regularly (periodic meetings about the EAAB, regular contact with tutors and PHU teams, use of the EAAB management system).

Finally, the Governability of the System category included four indicators intended to assess autonomy, adequate conditions, and articulation with other sectors so that the actors involved with the EAAB can develop appropriate activities and actions. The indicators measured in this category were coordination with other areas and/or spheres of government for the implementation of the EAAB; operationalization of the implementation of the EAAB; support to PHU for the development of actions (monitoring of BF and CF indicators and support in complying with the NBCAL); adherence of the actors involved.

To assess the organizational context, a qualitative questionnaire with questions covering the 17 indicators was given to the coordinator of the EAAB in the Federal District at the time. With the information about the proposed indicators, the answers were classified according to the influence they could exert on the implementation of the EAAB, as positive (facilitated the implementation of the EAAB) or negative (could hinder the implementation of the EAAB influence). The classification result was presented and validated by the manager in a virtual meeting.

#### 2.3.3. Evaluate the Degree of Implementation of the EAAB in the Health Units

To assess the degree of implementation of the EAAB in the PHUs, data were collected from all six certified PHUs in the FD. In these PHUs, previously tested questionnaires [19,20] were given to mothers of children aged zero to two years, health professionals from the PHU, and the managers of the PHU. The questionnaires included items that assessed compliance with the six EAAB certification criteria (described in the Introduction).

Questionnaire for mothers. Mothers of children born at term and not twins were eligible for the interview. The sample number of mothers to be included in the survey was estimated based on the number of children under two years of age who attended the Primary Health Care (PHC) in the PHUs in 2015 (information provided by an official letter from the Ministry of Health). To have district representation, adopting a conservative sizing of 50%, sampling error of 5%, and a confidence interval of 10%, the minimum sample size calculated was 152 mothers, distributed in the six PHUs with EAAB certification to evaluate its degree of implantation. The final sample consisted of 219 mothers, who were approached in the waiting room/hallway of the selected PHU on the day of their child’s growth and development consultation. A questionnaire included items on the mother’s health (prenatal care; routine consultations at the PHU) and aspects related to the child, such as BF and CF. The questionnaire also included items related to the EAAB certification criteria one and three.

Questionnaire for PHU professionals. This questionnaire was applied to one health professional in each PHU. To be included, the health professional had to be a doctor, nurse, or nutritionist and attend children in the growth and development follow-up appointments at the selected PHU (totaling 1 doctor and 5 nurses). Professionals were invited according to their availability on data collection days at the PHU. The questionnaire given to health professionals had items related to the organizational structure of the PHU, participation in training on BF and CF, flows of mothers’ care, and guidance and care given to pregnant women, mothers, and caregivers about BF and CF at the PHU. The questionnaire also included items related to the six criteria for EAAB certification.

Questionnaire for PHU managers. The sample included the six managers formally assigned to this function in each certified PHU. For managers, the questionnaire contained items related to the organizational structure of the PHU, professional training, and flows of service for mothers and children at the PHU. The questionnaire also included items related to the six criteria for EAAB certification.

In total, 3 types of questionnaires were used in the study, one for mothers, one for PHU professionals, and one for PHU managers, totaling 219 questionnaires for mothers, six questionnaires for PHU professionals, and six questionnaires for PHU managers. In addition, a structured interview was conducted with the coordinator of the EAAB in the Federal District, Brazil.

Triangulation of data. The analysis about the degree of EAAB implementation was carried out for each PHU, using the triangulation of information obtained from the questionnaires applied to mothers, professionals, and managers as well as items from the interview conducted with the manager of the EAAB in the FD, in the assessment stage for the organizational context. The degree of EAAB implementation in each certified PHU was evaluated as a global score from zero to 100 points.

To calculate this score, a consultation was conducted with specialists, since the number of items could vary by criterion and by respondent (mothers, professionals, managers, and coordinator of the EAAB in the FD). For example, Criterion one contemplated three items to be answered by mothers, five items by professionals, and five by managers. Eleven experts at the EAAB were invited to participate in this stage, professionals from universities, health services, and health departments, who know and have experience in the implementation of the EAAB in Brazil. Two rounds of consultations with specialists were conducted. In the first round, specialists classified the six EAAB certification criteria according to (a) order and degree of importance in the implementation of the EAAB, on a 5-point Likert scale (5 = very important, 4 = important, 3 = neutral, 2 = not very important, 1 = not at all important), and (b) importance of the respondent (mother, health professional, PHU manager, and the EAAB manager in the FD) in the evaluation of each EAAB implementation criterion. For this round, ten specialists responded. From the specialists’ answers, a scoring matrix of certification criteria for EAAB was constructed (Table 1), assigning a weighted score (out of 100 points) to each of the criteria. Subsequently, this score was divided and weighted, among the respondents, as a result of the consultation with specialists. Finally, this score was divided equally between the items of each respondent. In the second round, specialists validated the score by consensus, based on the Delphi methodology [31]. The degree of EAAB implementation in the PHU was classified as initial (up to 20 points), partial (21 to 50 points), advanced (51 to 80 points), and consolidated (81 points or over) [19].

**Figure 1:**
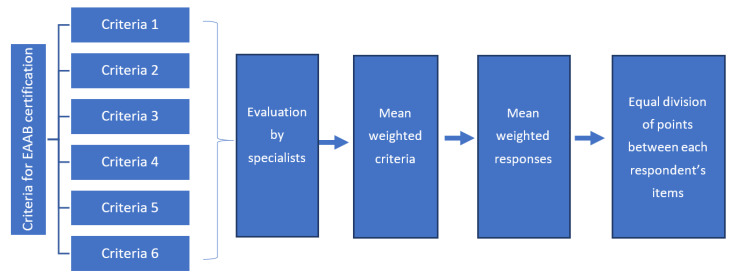
Flow of steps to calculate the weighted values of criteria and respondents to evaluate the degree of implementation of the EAAB in the Federal District, Brasília, 2021.

The steps for calculating the weighted values of the criteria and respondents for assessing the degree of implementation of the EAAB are shown in Figure 1.

#### 2.3.4. Analyze the Influence of the Organizational Context on the Degree of Implementation

The last stage of the analysis consisted of the convergence between the results of the degree of EAAB implementation in the PHUs and the indicators of the organizational context, to identify factors that can help or hinder the development of actions recommended by the six EAAB certification criteria.

## 3. Results

### 3.1. Logical Model of the EAAB Implementation

**Figure 2:**
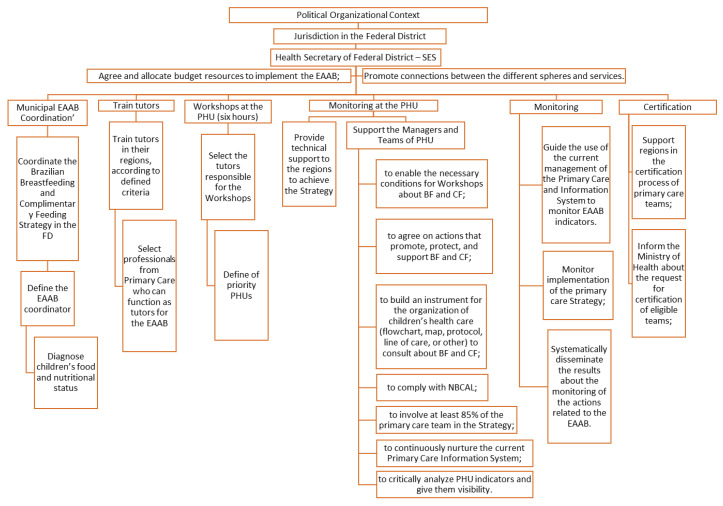
Logical model for the implementation of the EAAB in the Federal District.

A conceptual logical model of the EAAB in the FD was developed (Figure 2). The model enabled observation of the competencies and attributions related to the six stages identified for implementation of the EAAB in the FD: coordination of the EAAB, training of tutors, workshop at the PHU, follow-up at the PHU, monitoring, and certification. For each of the stages, the model indicated between one and three attributions, distributed among the actors involved, such as the municipal coordinator, tutor, managers, and teams from the PHUs. None of the stages identified a monitoring process for compliance by PHU with the criteria after certification. This conceptual logical model was used to interpret the organizational context and the degree of implementation of the EAAB in a PHU, as well as to identify gaps and weaknesses in the implementation stages of the EAAB in the FD.

### 3.2. Organizational Context of the EAAB in the Federal District

Of the seventeen indicators evaluated, ten were classified as positive influencers in the implementation of the EAAB in the FD (Table 2). Among the indicators evaluated in the Government Project category, three of the seven were classified as exerting a positive influence, such as having one District Policy on BF and the promotion in the PHC of actions to promote, protect, and support BF and healthy CF, positive reception of EAAB-maintained training of tutors with a higher workload even though the Ministry of Health had reduced this workload, and existence of activities, actions, and/or programs complementary to EAAB, such as a breastfeeding counseling course. However, four indicators had a negative influence: the lack of specific financial resources for the EAAB, the low Family Health Strategy (ESF) coverage in the PHC (less than 50%), PHU managers’ lack of interest in implementing the EAAB, and the competition of the EAAB with other priorities.

In the Governing Capacity category, five indicators were classified as a positive influence. The FD had a children’s health care area with a manager that had theoretical capacity and technical experience in conducting the EAAB, with a formal and appropriate relationship in the management organization chart. However, management technologies were not routinely used to monitor the EAAB, such as regular monitoring of BF rates.

In the System Governability category, half of the indicators (2) were classified as having a negative influence. The presence of cooperation with other areas and the operationalization of the actions foreseen by the EAAB must be highlighted; however, the support of the PHU for developing actions and the adhesion of the actors involved are still critical.

### 3.3. Degree of the EAAB Implementation in the Federal District

The implementation of the EAAB allowed identification of different scenarios between the PHUs in compliance with the certification criteria. Of the six PHUs certified by the EAAB in the FD, only one had a consolidated degree of implementation. The others (5) presented an advanced degree of implantation. Criteria five and six, which concern the participation of PHU teams (at least 85% of professionals) in the EAAB Workshop and the performance of BF and CF actions in the PHU Action Plan, are among the main problems in the implementation of the EAAB, with low percentages of compliance in the evaluated PHUs (Table 3).

### 3.4. Influence of Organizational Context on the Degree of Implementation

The analysis of the organizational context and the degree of implementation of the EAAB in the PHUs identified a negative influence of indicators in the three categories of the Triangle of Government related to difficulties in implementing criterion six (compliance with at least one BF action and one CF action), which had partial or incipient implementation in five of the six PHUs evaluated. For this criterion, the absence of specific resources for the EAAB, the fragility of the use of management technologies (such as the EAAB monitoring system), the lack of monitoring of food and nutrition indicators, and the adherence of involved actors, especially the PHU managers, were identified as factors that hinder the complete implementation of the EAAB. These same characteristics were identified as potentially interfering in the implementation of other criteria such as one, two, and five, which deal, respectively, with conducting systematic, individual, or collective actions to promote BF and healthy CF, monitoring of BF and CF indicators, and count on the participation of at least 85% of the team’s professionals in the developed workshops.

## 4. Discussion

Our study is one of the first to identify gaps in the stages of implementation of the EAAB that may compromise the expected impact on infant and young child feeding (IYCF) indicators and the organizational context that influences its implementation and sustainability. Among the six PHUs certified in the EAAB, all but one were found to have advanced degree of implementation. The PHUs analyzed had already achieved certification in 2014, which, at least in theory, indicates that they should have already met 100% of the criteria for EAAB implementation. During the period between certification and data collection for this research, no action to monitor the certification by the Brazilian Ministry of Health or the FD coordination was identified. Therefore, our analysis of the organizational context identified that the lack of specific funding and monitor systems to track BF and CF practices as well as certification compliance are the main challenges of EAAB sustainability in the FD.

Some aspects of the organizational context can facilitate the implementation of the EAAB. The indicators with positive influence include the existence of a specific district law that includes the EAAB actions as priorities, the positive receptivity of the EAAB by local management, and the existence of complementary actions for the EAAB (courses, events, and social mobilization campaigns). Other important positive influences are the existence of district coordination for the EAAB with professional experience in the subject, a public servant in the managerial position that has a function compatible with the management organization chart, and an operationalization of the EAAB implementation cooperating with other areas. A previous study that evaluated the strategy that preceded the EAAB also found that the existence of a municipal coordination sensitive to the proposed action facilitated the implementation across PHUs [19].

Despite an advanced implementation of the EAAB in certified PHUs in the FD, the context also elucidated unfavorable indicators that could have a negative influence. A critical point observed was the lack of specific resources for the implementation of the EAAB; despite the district law establishing that funded resources be allocated for the EAAB in the FD, in practice it was not happening. EAAB would receive funding from different resources when available and if available [15]. A study that analyzed the implementation of the EAAB in a capital city in Northeastern Brazil evidenced that budgetary resources are needed for the development of municipal activities proposed by the EAAB, such as the training workshops for tutors and resources to invest in establishing goals and partnerships [32]. Another work demonstrated that financial resources are also critical at the state management level (an area that is also the responsibility of the FD) to coordinate the implementation of the EAAB [31].

Other indicators of the organizational context that exerted a negative influence on the implementation of the EAAB are lack of management technologies employed (such as meetings with tutors and the use of monitoring systems) and not enough support for PHUs to develop actions that promote BF and CF. In the FD, the use of management technologies for monitoring the EAAB was deficient. Periodic meetings were not held, and few tutors entered information about holding workshops and monitoring of actions in the PHU regularly into the EAAB monitoring systems provided by the Ministry of Health. A recent study found that one of the barriers to implementing large-scale breastfeeding programs is a weak monitoring system [33]. Monitoring the implementation of EAAB at the state and the PHU level is critical to implementation success [31].

We found that monitoring of BF and CF indices has not occurred as recommended. A similar weakness in the organizational context of the EAAB was observed in a capital in the northeastern region of Brazil [32]. EAAB certification requires fulfillment of a group of six criteria. In criterion two, the monitoring of BF and CF indicators is required for certification and must be carried out by the current primary care system for this purpose, which at the national level is the Food and Nutritional Surveillance System (SISVAN) and more recently, the e-SUS [15,16]. Previous studies have shown that SISVAN is little used for food and nutrition surveillance and even less for planning intervention actions [34,35]. An evaluation of the precursor strategy of the EAAB, which also included the criterion of monitoring indicators of nutritional status and food consumption, found that monitoring of infant and nutrition indicators was weak among two out of three municipalities studied [20]. Furthermore, a recent study also found that monitoring of infant and young children feeding indicators is critical for successful implementation of the EAAB [33].

Other organizational context indicators with negative influence may also explain the low degree of consolidated implementation in PHUs certified by the EAAB in the FD. These indicators reflected the low interest of PHU managers associated with lack of understanding of the importance of the EAAB, competition of BF and CF with other health priorities, and challenges for adoption by actors that should be involved (e.g., PHU manager resistant to the EAAB proposal and tutors who are not released to carry out their activities), and barriers to conduct EAAB activities are examples of weaknesses related to important aspects of its implementation. Our findings corroborate with prior analysis that identified the importance of the commitment of teams to the action plan and proposed actions to increase support to BF and CF, support for the role of the tutor, training of teams, and prioritization of the EAAB in a Municipal Health Plan [21,22].

It is worth noting that, even though it was not evaluated in this study, some findings from other studies have already shown that PHUs that implemented actions or were certified in the EAAB, or in programs similar to the EAAB, had higher prevalence of BF when compared to PHUs without the programs [13,21,36,37]. A study carried out in a municipality in São Paulo, Brazil showed that, in children treated at PHUs certified by the Brazil Breastfeeding Network, the prevalence of BF was higher when compared to noncertified PHUs [36]. However, another study also showed that certification in the Brazil Breastfeeding Network program alone was not a guarantee of a higher prevalence of BF, identifying that BF was higher in children attending PHUs that met all the certification criteria required in the program when compared to certified PHUs that did not meet all criteria [37].

Our findings pointed to challenges in the sustainability of the EAAB in the FD, considering that the study evaluated only certified PHUs, where all were expected to have a consolidated degree of implementation of the certification criteria. Although most PHUs certified in the FD only exhibited advanced but not consolidated implementation, the result may have been facilitated by an organizational context that reinforces actions of the EAAB through a district law and is managed by an active coordinator in the FD who is in a stable employment position and has accumulated technical competence in the subject. Furthermore, the cooperation with other areas, as well as the actions and activities that complement the EAAB, such as other courses and professional training about BF, probably strengthened this scenario. However, the consolidation of the EAAB in the FD must still be strengthened, by including earmarked funding, regular use of management technologies to monitor actions, prioritization of the EAAB in the face of other health priorities demands, and the support of PHU managers, professionals in the PHU, and tutors.

This study has some limitations to be considered when interpreting the findings. This study had a favorable bias towards the degree of implementation, considering that it evaluated only PHUs certified by the EAAB. Nevertheless, the implementation analysis made by multiple tools, such as the construction of a specific conceptual logical model for the EAAB in the FD, the identification of organizational context analysis indicators, and the construction of an EAAB certification criteria score matrix duly validated by specialists with experience in the implementation of the EAAB, proved to be an important tool for managers to define strategies for evaluating a PHU after certification, which was an important gap identified here. Furthermore, we analyzed information from multiple actors such as mothers, professionals, PHU managers, and the EAAB manager in the FD, allowing a triangulation of information, which provided robustness to the results found in this study. However, excluding PHUs that started implementation but did not achieve certification and analyzing only certified PHUs limited the scope of analysis on the implementation of the EAAB. Lessons learned from our research can inform the planning of organizational contexts and investment of public health funding when implementing an IYCF at a large scale, given that it contributes to the establishment of methodologies that identify critical factors for the implementation of breastfeeding and complementary feeding programs and that direct public policy managers to invest in the development of sustainable programs.

## 5. Conclusions

All but one of the Primary Health Units certified by the EAAB in the Federal District presented advanced implementation, close to what is recommended by the Brazilian Ministry of Health; however, challenges remain for its consolidation. Lack of specific funding and monitor systems to track breastfeeding and complementary feeding practices as well as certification compliance are the main challenges of EAAB sustainability in the Federal District.

## Figures and Tables

**Table 1:** Scoring matrix of certification criteria for the EAAB in the Federal District, Brasília, 2018.

			Respondent							
			Mother		Professional		Manager		Coordinator	
N° Criterion	Criterion Name	Score on the Criterion	N° of Items	Score on Item	N° of Items	Score on Item	N° of Items	Score on Item	N° of Items	Score on Item
1	Develop individual or collective systematic actions to promote breastfeeding and complementary feeding	18	3	2.3	5	1.2	5	1		
2	Monitor breastfeeding and complementary feeding rates	18			1	7	1	6	1	5
3	Provide an instrument for child health care organization (flowchart, map, protocol, line of care, or other) to detect problems related to breastfeeding and complementary feeding	16	7	0.57	8	0.75	9	0.66		
4	Comply with the Brazilian Guidelines for the Marketing of Food for Infants and Young Children, Nipples, Pacifiers, and Bottles (NBCAL) and with Law No. 11,265 of 2006, and do not distribute “substitutes” for breast milk at the PHU	16			5	1.2	5	1.2	1	4
5	Count on the participation of at least 85% of the team’s professionals in the developed workshops	17			1	9	1	8		
6	Fulfill at least one breastfeeding action and one complementary feeding action established in the action plan	15			3	2.66	3	2.3		
	TOTAL	100								

**Table 2:** Classification of indicators in the categories of Government Triangle, EAAB, Brasília, FD, 2018.

Categories	Indicators	Federal District
Government Project	Primary Care as a priority—ESF Coverage ^1^	less than 50%
	Breastfeeding and complementary feeding as a priority in policy/legislation or guideline ^2^	Law 5374/2014 about the District Policy on BF and the promotion in primary health care of actions to promote, protect, and support breastfeeding and healthy complementary feeding.
	Interest in implementing the EAAB ^1^	“The PHU manager needs to ‘buy into the idea’, be a partner. He needs to be convinced of the importance of the EAAB for the PHU and the population.”
	Receptivity of EAAB in the governmental sphere ^2^	“Positive reception. They tried to adapt but maintain the 40 h for the tutor training workshop at EAAB. They left more time for discussion (maintained the training of tutors with a higher workload even though the Ministry of Health had reduced it)”
	Competition of EAAB with other priorities ^1^	“The health system still works on the logic of disease and not health promotion. The PHU manager is more concerned with spontaneous demand than with health promotion activities for breastfeeding and healthy complementary feeding. The PHU manager needs to understand that the EAAB is important.”
	Existence of activities, actions, and/or programs complementary to EAAB ^2^	40-hour breastfeeding counseling course (held in May and August since 2016) in all health regions. A total of 132 professionals were trained in the first semester of 2019. Trains professionals from State Secretary of Health of Federal District (SES), universities, and the supplementary network. Mobilization of Breastmilk Donation Day, District Law for Breastmilk Donation Week, District Law for Golden August. Mobilization of Golden August. Opening of events in partnership with the judicial system, breastfeeding seminars (since 2016). Two seminars for two groups (650 participants in 2018). May: communication mobilization, Amamenta Brasília website, Facebook, application, breast milk donation system, telephone, local events in all HMBs; in August, mobilization in the 7 health regions, where there are HMBs in the region, also participates in the mobilization of World Breastfeeding week (WBW). D-day of breastfeeding. Discussion about NBCAL at an event, film screenings, seminars. 40-hour breastfeeding courses for primary care.
	Specific financial resource for EAAB ^1^	“There is no specific resource. Funds from lawmakers. SES Funding. Stork Network resource, own resources of SES workers.”
Government Capacity	Existence of an area for Children’s Health ^2^	Coordination of Breastfeeding Policies in the FD
	Existence of EAAB Coordinator or breastfeeding and complementary feeding actions ^2^	“Yes, coordinator of breastfeeding policies for the FD”
	Stability of the person responsible for breastfeeding actions and healthy complementary feeding (bonded) ^2^	Public servant
	Professional experience of the person responsible for breastfeeding and healthy complementary feeding actions ^2^	11 years of experience in the position coordinating breastfeeding policies, technical experience in the subject of children’s health, breastfeeding, and complementary feeding.
	Institutional functions of EAAB managers compatible with the organizational chart ^2^	Pediatrician—coordinator of the breastfeeding policies in the FD
	Use of management technologies (periodic meetings about the EAAB, regular contact with tutors and PHU teams, use of the EAAB management system) ^1^	“There were monthly meetings that no longer happen with the change in the structure of the SES. Access the EAAB system but reports that few tutors enter information.”
System Governability	Coordination with other areas and/or spheres of government to implement the EAAB ^2^	“There is coordination with the Board of the Family Health Strategy and GESNUT (Management of Nutrition Services).”
	Operationalization of the implementation of the EAAB ^2^	Workshop to train tutors trying to cover all health regions, tutors with profiles became workshop facilitators, and others were only trained without acting as tutors.
	Support to PHU for the development of actions (monitoring of BF and CF indicators, compliance with NBCAL) ^1^	Professional training calendar “Counseling–40 h in May and August (primary care, supplementary, and university) by region. B-course taken in hospitals—24 h (hospital management and counseling) NBCAL—course for professionals (1 course in the last three years) BFHI and CF course in 2018 for breastfeeding professionals. “The monitoring data of breastfeeding and complementary feeding indicators come from surveys (prevalence of 2008), 2014-pilot by telephone. 2016/2017–Project “Early Childhood for Healthy Adults” (PIPAS). e-SUS (entered as an electronic medical record in PHC in 2017/2018, but there is still resistance in filling in part of the PHU servers. The Health Regions must have followed the indicators of compulsorily BF by the SES since 2010. Collection of human milk is also monitored by the SES.” “Local data from recent dietary prevalence surveys? Surveys (2008 prevalence), 2014–telephone pilot. 2016/2017–PIPAS” On the distribution of milk and baby formula in the PHU: “No. Only in the STD/AIDS Reference Units (HIV and HTLV patients)–screening since 2012, an average of 35 women per year diagnosed with HTLV. Children with food allergy (central pharmacy).”
	Adherence of the actors involved ^1^	“Tutor is not institutional; the manager does not always allow the professional to do the activities of a tutor. Complaint of the tutor “how can the tutor’s workload be made official?” “The PHU manager needs to ‘buy into the idea’(resistant to the EAAB proposal), be a partner. He needs to be convinced of the importance of the EAAB for PHUs and the population.”

^1^ Negative influence. ^2^ Positive influence.

**Table 3:** Percentage of compliance with EAAB criteria and classification in the implementation score, Brasília, FD, 2018.

	PHU 1	PHU 2	PHU 3	PHU 4	PHU 5	PHU 6
Criteria 1	61.7 ^2^	61.7 ^2^	61.7 ^2^	74.4 ^2^	49.4 ^3^	61.7 ^2^
Criteria 2	66.7 ^2^	61.1 ^2^	27.8 ^3^	100 ^1^	100 ^1^	100 ^1^
Criteria 3	78.7 ^2^	78.7 ^2^	61.2 ^2^	78.1 ^2^	82.5 ^1^	78.7 ^2^
Criteria 4	85 ^1^	100 ^1^	100 ^1^	85 ^1^	85 ^1^	70 ^2^
Criteria 5	0 ^4^	100 ^1^	100 ^1^	100 ^1^	0 ^4^	100 ^1^
Criteria 6	46.7 ^3^	100 ^1^	34 ^3^	0 ^4^	0 ^4^	46.7 ^3^
TOTAL	56.3 ^2^	82.7 ^1^	63.9 ^2^	74.6 ^2^	53.8 ^2^	76.9 ^2^

^4^ Initial implementation. ^3^ Partial implementation. ^2^ Advanced implementation. ^1^ Consolidated implementation.

## Data Availability

The data presented in this study are available on request from the corresponding author. The data are not publicly available as they contain information that may violate the privacy of research participants.
