# Correction: Moura et al. Implementation of the Strategy for Breastfeeding and Complementary Feeding in the Federal District in Brazil. *Int. J. Environ. Res. Public Health* 2022, *19*, 5003

**DOI:** 10.3390/ijerph20166594

**Published:** 2023-08-18

**Authors:** Amanda Souza Moura, Muriel Bauermann Gubert, Sonia Isoyama Venancio, Gabriela Buccini

**Affiliations:** 1 Department of Nutrition, Center of Epidemiological Studies of Health and Nutrition (NESNUT), Faculty of Health Sciences, University of Brasília, Brasília 70910-900, Brazil; murielgubert@gmail.com; 2 Institute of Health, State Secretariat of São Paulo Health, São Paulo 01314-000, Brazil; soniavenancio@uol.com.br; 3 Department of Social and Behavioral Health, University of Nevada, Las Vegas, NV 89119, USA; gabriela.buccini@unlv.edu

The authors would like to make the following corrections to this paper [1].

In the original article, there was a mistake in Table 2. In the legend of Table 2, the indicators of a negative influence correspond to the number 1 and the indicators of a positive influence correspond to the number 2. The correct version of Table 2 appears below.

The authors state that the scientific conclusions are unaffected. This correction was approved by the Academic Editor. The original publication has also been updated.

## Figures and Tables

**Table 2:** Classification of indicators in the categories of Government Triangle, EAAB, Brasília, FD, 2018.

Categories	Indicators	Federal District
Government Project	Primary Care as a priority—ESF Coverage ^1^	less than 50%
	Breastfeeding and complementary feeding as a priority in policy/legislation or guideline ^2^	Law 5374/2014 about the District Policy on BF and the promotion in primary health care of actions to promote, protect, and support breastfeeding and healthy complementary feeding.
	Interest in implementing the EAAB ^1^	“The PHU manager needs to ‘buy into the idea’, be a partner. He needs to be convinced of the importance of the EAAB for the PHU and the population.”
	Receptivity of EAAB in the governmental sphere ^2^	“Positive reception. They tried to adapt but maintain the 40 h for the tutor training workshop at EAAB. They left more time for discussion (maintained the training of tutors with a higher workload even though the Ministry of Health had reduced it)”
	Competition of EAAB with other priorities ^1^	“The health system still works on the logic of disease and not health promotion. The PHU manager is more concerned with spontaneous demand than with health promotion activities for breastfeeding and healthy complementary feeding. The PHU manager needs to understand that the EAAB is important.”
	Existence of activities, actions, and/or programs complementary to EAAB ^2^	40-hour breastfeeding counseling course (held in May and August since 2016) in all health regions. A total of 132 professionals were trained in the first semester of 2019. Trains professionals from State Secretary of Health of Federal District (SES), universities, and the supplementary network. Mobilization of Breastmilk Donation Day, District Law for Breastmilk Donation Week, District Law for Golden August. Mobilization of Golden August. Opening of events in partnership with the judicial system, breastfeeding seminars (since 2016). Two seminars for two groups (650 participants in 2018). May: communication mobilization, Amamenta Brasília website, Facebook, application, breast milk donation system, telephone, local events in all HMBs; in August, mobilization in the 7 health regions, where there are HMBs in the region, also participates in the mobilization of World Breastfeeding week (WBW). D-day of breastfeeding. Discussion about NBCAL at an event, film screenings, seminars. 40-hour breastfeeding courses for primary care.
	Specific financial resource for EAAB ^1^	“There is no specific resource. Funds from lawmakers. SES Funding. Stork Network resource, own resources of SES workers.”
Government Capacity	Existence of an area for Children’s Health ^2^	Coordination of Breastfeeding Policies in the FD
	Existence of EAAB Coordinator or breastfeeding and complementary feeding actions ^2^	“Yes, coordinator of breastfeeding policies for the FD”
	Stability of the person responsible for breastfeeding actions and healthy complementary feeding (bonded) ^2^	Public servant
	Professional experience of the person responsible for breastfeeding and healthy complementary feeding actions ^2^	11 years of experience in the position coordinating breastfeeding policies, technical experience in the subject of children’s health, breastfeeding, and complementary feeding.
	Institutional functions of EAAB managers compatible with the organizational chart ^2^	Pediatrician—coordinator of the breastfeeding policies in the FD
	Use of management technologies (periodic meetings about the EAAB, regular contact with tutors and PHU teams, use of the EAAB management system) ^1^	“There were monthly meetings that no longer happen with the change in the structure of the SES. Access the EAAB system but reports that few tutors enter information.”
System Governability	Coordination with other areas and/or spheres of government to implement the EAAB ^2^	“There is coordination with the Board of the Family Health Strategy and GESNUT (Management of Nutrition Services).”
	Operationalization of the implementation of the EAAB ^2^	Workshop to train tutors trying to cover all health regions, tutors with profiles became workshop facilitators, and others were only trained without acting as tutors.
	Support to PHU for the development of actions (monitoring of BF and CF indicators, compliance with NBCAL) ^1^	Professional training calendar “Counseling–40 h in May and August (primary care, supplementary, and university) by region. B-course taken in hospitals—24 h (hospital management and counseling) NBCAL—course for professionals (1 course in the last three years) BFHI and CF course in 2018 for breastfeeding professionals. “The monitoring data of breastfeeding and complementary feeding indicators come from surveys (prevalence of 2008), 2014-pilot by telephone. 2016/2017– Project “Early Childhood for Healthy Adults” (PIPAS). e-SUS (entered as an electronic medical record in PHC in 2017/2018, but there is still resistance in filling in part of the PHU servers. The Health Regions must have followed the indicators of compulsorily BF by the SES since 2010. Collection of human milk is also monitored by the SES.” “Local data from recent dietary prevalence surveys? Surveys (2008 prevalence), 2014–telephone pilot. 2016/2017–PIPAS” On the distribution of milk and baby formula in the PHU: “No. Only in the STD/AIDS Reference Units (HIV and HTLV patients)–screening since 2012, an average of 35 women per year diagnosed with HTLV. Children with food allergy (central pharmacy).”
	Adherence of the actors involved ^1^	“Tutor is not institutional; the manager does not always allow the professional to do the activities of a tutor. Complaint of the tutor “how can the tutor’s workload be made official?” “The PHU manager needs to ‘buy into the idea’(resistant to the EAAB proposal), be a partner. He needs to be convinced of the importance of the EAAB for PHUs and the population.”

^1^ Negative influence. ^2^ Positive influence.
